# A High Resolution Melting Analysis-Based Genotyping Toolkit for the Peach (*Prunus persica*) Chilling Requirement

**DOI:** 10.3390/ijms21041543

**Published:** 2020-02-24

**Authors:** Lin Chou, Shih-Jie Huang, Chen Hsieh, Ming-Te Lu, Chia-Wei Song, Fu-Chiun Hsu

**Affiliations:** 1 Department of Horticulture and Landscape Architecture, National Taiwan University, Taipei 10617, Taiwan; choulin@gate.sinica.edu.tw (L.C.); r06628112@ntu.edu.tw (S.-J.H.); r06628114@ntu.edu.tw (C.H.); 2 Crop Science Division and Guansi Experiment Station, Taiwan Agricultural Research Institute, Council of Agriculture, Taichung 41301, Taiwan; mingtelu@tari.gov.tw (M.-T.L.); pcsung@tari.gov.tw (C.-W.S.)

**Keywords:** chilling requirement (CR), genotyping, high-resolution melting (HRM) analysis, marker-assisted selection (MAS), principal component analysis (PCA), quantitative trait loci (QTLs), single nucleotide polymorphisms (SNPs)

## Abstract

The chilling requirement (CR) is the main factor controlling the peach floral bud break and subsequent reproductive growth. To date, several peach CR quantitative trait loci (QTLs) have been identified. To improve the accessibility and convenience of this genetic information for peach breeders, the aim of this study was to establish an easy-to-use genotype screening system using peach CR molecular markers as a toolkit for marker-assisted selection. Here, we integrated 22 CR-associated markers from three published QTLs and positioned them on the *Prunus persica* physical map. Then, we built a PCR-based genotyping platform by using high-resolution melting (HRM) analysis with specific primers and trained this platform with 27 peach cultivars. Due to ambiguous variant calls from a commercial HRM software, we developed an R-based pipeline using principal component analysis (PCA) to accurately differentiate genotypes. Based on the PCA results, this toolkit was able to determine the genotypes at the CR-related single nucleotide polymorphisms (SNPs) in all tested peach cultivars. In this study, we showed that this HRM-PCA pipeline served as a low-cost, high-throughput, and non-gel genotyping solution. This system has great potential to accelerate CR-focused peach breeding.

## 1. Introduction

The chilling requirement (CR) is a characteristic that evolved in deciduous fruit tree species, such as peach trees (*Prunus persica*), and is a measure of the period of time during which trees withstand low temperatures to avoid flowering in winter. The CR duration is the major climatic adaptation that limits the geographic distribution in which an individual tree can flower [1]. To effectively increase the distribution of peach cultivation regions from temperate zones, it is necessary to breed new cultivars with low CRs that are better adapted to subtropical or tropical regions [2,3]. Furthermore, it is important to breed peach cultivars with low CRs given that global climate change has contributed to insufficient chill accumulation that has resulted in decreased peach production [2,4]. However, peach breeding to produce low CRs requires years of cultivation for individuals to reach the adult stage, and CR phenotyping requires multiple years of observation of cumulative chilling and blooming dates. Additionally, the CR in peach trees is known to be a polygenic and heritable trait [5,6] with high broad-sense heritability (e.g., H^2^ = 79.5%) [7]. The characteristics of the CR increase the complexity of breeding for CR traits. Therefore, comprehensive genetic CR studies are needed that provide the necessary knowledge to improve breeding efficiency and adapt peach varieties to future climatic conditions.

Three current studies have made major genetic breakthroughs by mapping the quantitative trait loci (QTLs) associated with CRs and blooming dates [7,8,9,10]. The first study included 378 individuals of an F_2_ population derived from the parental cultivars ‘Contender’ × ‘Fla.92-2C’ with high- and low-CR, respectively, for QTL mapping [7]. Four QTLs were found to be responsible for controlling the CR. This study also utilized two low-CR and two high-CR individuals from the F_2_ population for whole genome sequencing to generate a list of candidate genes associated with the QTLs, the intervals of which were represented using 18 simple sequence repeat (SSR) markers [8]. Two independent QTL studies used similar bi-parental approaches on two different progeny populations [9,10]. Briefly, 12 QTLs controlling the CR and six representative single nucleotide polymorphisms (SNPs) were identified in the ‘V6’ × ‘Granada’ F_2_ population [9]. The third study utilized the genotyping-by-sequencing (GBS) method to identify nine SNPs and one SSR representing 10 QTLs from the ‘Hakuho’ × ‘UFGold’ F_2_ population [10]. These mapping results have contributed positional genetic data and have provided important information for further functional genomic research. Integrating the positions of these QTLs that were identified from each study is important to determine the overlapping QTL region.

These CR-related QTLs are promising in terms of genetic mapping, but the development of convenient breeding tools is required to break the technical entry barriers associated with the use of marker-assisted selection (MAS) [11]. SSR markers can be detected using polymerase chain reaction (PCR)-based techniques with common lab equipment, which is accessible for most breeders. Eighteen SSRs have been used to indicate eight CR-related QTL intervals [8], but these SSR markers only cover a portion of the reported peach CR-related QTLs [7,8,9,10]. Although SSR markers are highly accessible and cost effective, the efficiency of such gel-based and laboratory-intensive approaches may be greatly reduced when the number of samples or markers increases.

A shift in molecular marker approaches from SSR markers to SNP markers has been observed in this decade [12]. The release of the peach reference genome has facilitated the exploration of SNPs from broader genetic pools using genome-wide sequencing and has further developed the SNP array [13,14,15]. As far as peach SNP-based mapping goes, GBS has been utilized to identify SNPs for the construction of linkage maps and to identify QTLs associated with CRs and blooming dates [10]. The peach 9K Infinium II array [13] has been used to genotype SNPs for QTL mapping of the CRs, flowering times, heat requirements, and ecodormancy releases [9]. These sequencing- and array-based approaches are the high-throughput and multiplex platforms. These platforms are able to sequence multiple samples in a single Illumina HiSeq lane and/or to identify thousands of SNPs simultaneously for each sample in an array. Nevertheless, data management, bioinformatic platforms, and analytical tools are necessary to convert sequence information to SNPs [11]. Since a 9K SNP array would generate extra data that are not needed for MAS and the array approach is not designed for targeting particular traits in a MAS when the sample number is large, the development of an accessible and economical MAS platform to detect peach SNPs for CR-related QTLs is important.

The detection of SNPs can be accessible for common labs when various gel-free fluorescence-based methods are employed, including TaqMan® assays, kompetitive allele-specific PCR (KASP) assays, and high-resolution melting (HRM) analysis. The TaqMan® assay utilizes two PCR primers and a dual-labelled allele-specific probe [16,17]. KASP uses three unlabeled primers in combination with two universal fluorescence resonance energy transfer (FRET) quenching reporters [18]. HRM only requires two allele-specific primers for PCR and the melting behavior of the PCR products is used to discriminate SNP variance [19,20]. Given that HRM and KASP do not required fluorophore-labelled allele-specific oligonucleotides and that HRM and KASP reactions can be carried out with universal master mixes and universal FRET cassettes, respectively, these two methods are sufficiently flexible and cost-effective to be adopted by breeders. Current studies have successfully utilized HRM for high-throughput plant genotyping [21,22,23], cultivar identification, and the authenticity analysis of food products [24,25,26]. Considering that HRM only requires two short primers, the cost of HRM is likely to be a little bit lower than that of KASP. Advanced statistical methods for HRM analysis are available to improve variant calling [27], and using HRM for plant genotyping may be a feasible approach.

The establishment of a peach breeding toolbox is usually initiated from mapped genes or loci that have been highly linked with horticulture traits, followed by the identification of specific genetic markers according to genetic position for the implementation of further MAS. Major genes or markers that are highly linked with several qualitative peach traits have been identified. For example, a carotenoid cleavage dioxygenase gene has been characterized and shown to control for yellow or white fruit flesh color [28,29]. Two genes encoding endopolygalacturonases (endoPGs) have also been reported to be candidates that control traits related to stone adhesion and flesh texture [30,31]. Furthermore, the difference between peaches and nectarines is due to fruit skin pubescence, which is controlled by a MYB transcription factor [32]. In addition, qualitative peach resistance to the green peach aphid is conferred by an *Rm2* dominant allele that encodes a member of the nucleotide binding site leucine-rich repeat (NBS-LRR) resistance protein family [33]. Lastly, flat and round peach fruit shapes are linked with an SSR marker, UDP98-412, that is present in most flat fruit cultivars [34]. For the implementation of MAS, the association of fruit shape traits is further improved by the use of a haplotype that is represented with 3 SNPs or by a primer pair for two small indels [35]. Notably, the peach blush trait, the color red on the fruit skin surface, is able to be routinely predicted using a 5-SNP haplotype test in a single PCR-based assay [36]. The potential implementation of high-throughput MAS has been further developed based on the positional information for these multiple qualitative traits using peach SNP arrays [37,38] or GBS [39].

To facilitate peach cultivar breeding with the desired CR traits, we aimed to establish an easy-to-use genotyping platform with which plant breeders can develop new peach cultivars by genotyping the representative QTL markers that control the CR. In our study, the publicly available whole genome sequences in the Genome Database for Rosacea [40] were used as a physical reference to integrate published CR-related QTLs, and HRM-optimized primer pairs were designed to be specific to each major-effect QTL. To improve the genotyping accuracy for HRM data, we developed a free and open source R-based pipeline for variance calling. A genotyping toolkit that consists of these primer sets and a pipeline for variant calling will allow breeders to determine the genotypes of the SNP markers that are linked to the CR-related QTLs.

## 2. Results

### 2.1. Integration of Genetic Cofactors Representing Major- and Minor-Effect CR-related QTLs on a Physical Peach Genome Map

To situate the genetic cofactors representing the CR-related QTLs onto a physical map, we took advantage of the *Prunus persica* genome v.2.0.a1 [14], using it as the physical reference with which to position the cofactors based on SSR allele-specific primer sequences or SNP marker sites that were used for QTL mapping in previous studies [8,9,10]. All cofactors were assigned to represent either peach CR major-effect QTLs (Table 1) or minor-effect QTLs (Appendix A Table A1), according to the statistics and descriptions of the original QTL mapping studies [8,9,10].

Briefly, 6 SNP markers, including SNP_IGA_122057, SNP_IGA_779224, SNP_IGA_769194, SNP_IGA_297497, SNP_IGA_293752, and SNP_IGA_635355, were filtered from 12 QTLs in ‘V6’ × ‘Granada’ F_2_ progeny [9], according to the logarithm of the odds (LOD) score and the percentage of phenotypic variance of these QTLs. With similar criteria, 12 SSR markers (i.e., Pchgms29, Pchgms174, UDAp-460, UDAp-409A, UDA-053, BPPCT036B, Pchgms170, AMPA103, M12a, ssrPACITA21, EPPISF002, and PacC13) were selected for 17 QTLs from the ‘Contender’ × ‘Fla.92-2C’ F_2_ population [8], and 10 QTLs containing 9 SNP markers and 1 SSR marker (i.e., 1_40995799, 1_44762763, 4_14984691, 4_13747914, 2_16900230, 4_00772820, 4_11060745, 5_13713689, BPPCT038, and 8_11718744) were chosen from a study using the ‘Hakuho’ × ‘UFGold’ F_2_ population [10]. Taken together, the 28 cofactors linked to the QTLs controlling the CR were integrated into a physical map. To further classify these QTLs into major- or minor-effect QTLs, their degree of influence on the CR was used by setting a threshold for each QTL study. Around 10–20% of the phenotypic variance was set as the cut-off threshold to select major-effect QTL candidates. These candidates were further assessed individually by considering the LOD scores and presence of over-lapping/nearby QTLs (Table 1 and Appendix A Table A1).

### 2.2. Selection of SNP Markers Presenting Cofactors and the Detection of the Selected SNPs via HRM Analysis

For the establishment of a breeder-accessible genotyping system, cofactors were surveyed by detecting their flanking SNPs via HRM analysis. In order to increase the uniformity of the HRM analysis, all cofactors linked with the SSR markers and some SNP markers were replaced with adjacent SNP markers (i.e., selected markers). To perform HRM genotyping, specific primer pairs with a near—100% PCR efficiency were designed for all selected markers (Appendix A Table A2). The performance of HRM using these primer pairs was tested by polymorphism genotyping in our collection of 27 peach cultivars, including 15 low-chill cultivars and 12 high-chill cultivars. Nonetheless, no suitable primers were designed among the 28 cofactors that were able to differentiate the genotypes of the four cofactors linked to the four minor-effect QTLs in the genomic region, including SNP_IGA_293752, ssrPACITA21, PacC13, and BPPCT038 (Appendix A Table A1). In addition, no polymorphisms were detected on the flanking SNPs of two other minor-effect cofactors, Pchgms170 and SNP_IGA_635355, in any of the 27 tested peach cultivars. Considering that the influence of these minor-effect cofactors on the observed phenotypes was relatively low, these six cofactors were excluded from subsequent experiments. In summary, a total of 22 SNP markers were selected that were comprised of 11 major-effect (Table 1) and 11 minor-effect (Appendix A Table A1) QTLs.

**Figure 1:**
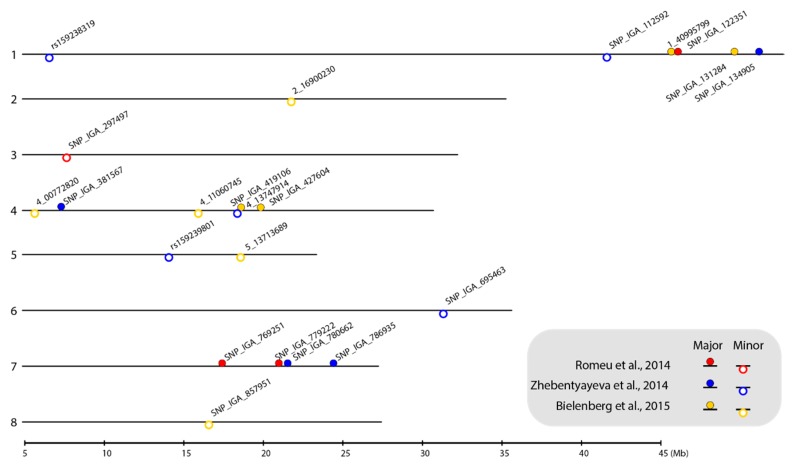
Location of selected SNP markers linked to chilling requirement (CR)-related QTLs on the *P. persica* genome v.2.0.a1 physical map. The numbers on the left side of the bars indicate the linkage group (LG). The selected SNP markers are shown as closed circles (markers linked to major-effect QTLs) and open circles (markers linked to minor-effect QTLs). Circles filled with red, blue, and yellow represent the markers identified from the ‘V6’ × ‘Granada’ [9], ‘Contender’ × ‘Fla.92-2C’ [8], and ‘Hakuho’ × ‘UFGold’ [10] F_2_ populations, respectively. The map was plotted using the genoPlotR package in RStodio [41,42].

Using the *P. persica* genome v.2.0.a1 [40] as a reference, we positioned all 22 SNP markers on the physical map to represent the locations of the major- and minor-effect CR-related QTLs (Figure 1). Notably, the SNPs linked to the major-effect QTLs were mainly located in LG1, LG4, and LG7 and the markers linked to the minor-effect QTLs were scattered on seven out of a total of eight linkage groups (LGs) (Figure 1). In addition, the farthest physical distance between the original cofactors and the selected SNP markers was 20 kbp and most were located within 10 kbp of each other (Table 1 and Appendix A Table A1), indicating that the recombination rate between these cofactors and the SNP marker pairs was very low.

**Figure 2:**
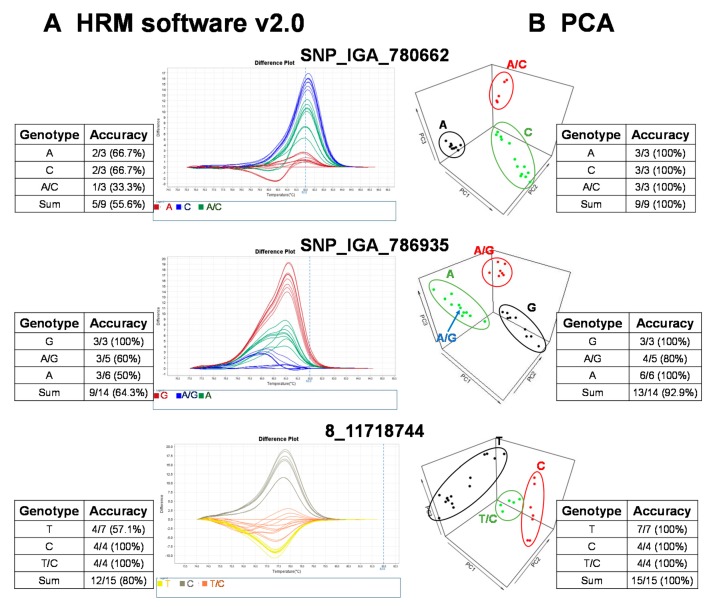
Validation of high resolution melting (HRM) analysis for three representative SNP markers in 27 peach cultivars followed by variant calling using a commercial software, HRM v. 2.0, and an R-based principal component analysis (PCA) with variant validation by Sanger sequencing. The genotyping results grouped with (**A**) HRM software v. 2.0 (Applied Biosystems, Waltham, MA, USA) and (**B**) the PCA pipeline. The validation of the HRM detection results was conducted with Sanger sequencing. The accuracy of HRM software or PCA results was calculated as follows: the number of variant calls consistent with Sanger sequencing/total sample number sequenced with Sanger sequencing.

The raw fluorescence data from the 22 SNP markers of the HRM analysis were analyzed with the commercial software, High Resolution Melt Software v. 2.0 (Applied Biosystems, Waltham, MA, USA). The temperature regions of the melt curves, such as the pre-melt and post-melt regions, that were used for each primer pair are listed in Table 2. In each of the difference plots, the curves of the 27 cultivars were clustered into 2–3 variants based on the reassembled melt curve patterns (Figure 2). Cultivars in the same cluster were assumed to share the same genotypes for the given SNP marker. To assess the genotypes of the SNP targets, at least three cultivars from each cluster were randomly selected for Sanger sequencing. The sequencing results showed that some cultivars in the same cluster possessed different genotypes (Figure 2). As an example, the accuracy of three marker (i.e., SNP_IGA_780662, SNP_IGA_786935, and 8_1171818744) variant calls from HRM Software v. 2.0 (Applied Biosystems, Waltham, MA, USA) was 55.6%, 64.3%, and 80%, respectively (Figure 2). To determine whether this low observed accuracy of the two SNPs was attributed to either the HRM reactions or the use of HRM Software v. 2.0 (Applied Biosystems), the reproducibility of the HRM reactions was assessed by carrying out independent HRM reactions for correctly and incorrectly genotyped samples. After the subsequent analysis of these samples with HRM Software v. 2.0 (Applied Biosystems), we concluded that it is likely that the observed low accuracy was due to the use of HRM Software v. 2.0 (Applied Biosystems) since both the correctly and incorrectly genotyped samples were reproducible in multiple independent experiments. Thus, an alternative analysis method to improve the variant call was required.

### 2.3. HRM Followed by Principal Component Analysis (PCA) is a Robust Variant Calling Method for Differentiating Genotypes Based on the Selected SNP Markers

To provide a robust method to call variants for HRM analysis, we established a free and open source R-based pipeline to execute variant calling with PCA, based on the rationale of a previous study [27]. This study claimed regular HRM software uses the shape of melting curves that are not supported by statistics, and suggested automated statistical methods such as PCA was more appropriate [27]. In our simplified version, the normalization of the raw fluorescence data for HRM analysis was carried out using the optimized lower limit (pre-melt region) and upper limit (post-melt region) temperatures of the melt curve analysis for each primer pair (Table 2). All peach cultivars were grouped into 2–3 clusters, and we randomly selected some cultivars from each cluster for Sanger sequencing to validate the genotypes. As a result, the prediction accuracy was significantly increased with the PCA pipeline method compared to that of the results produced by HRM Software v. 2.0 (Figure 2). For example, the genotyping accuracy of SNP_IGA_780662 and 8_11718744 increased from 55.6% and 80% to 100%, respectively, and the accuracy of SNP_IGA_786935 increased from 64.3% to 92.9% when the PCA clustering method was applied (Figure 2). These results support the conclusion that the developed R-based PCA script, which is free to use, is a highly reliable tool for HRM variant calling.

### 2.4. Genotyping of 22 CR-related SNP Markers for 27 Peach Cultivars

The PCA pipeline was subsequently used to determine the genotypes of all the selected SNP markers, including the 11 major-effect markers (Table 3) and the 11 minor-effect markers (Appendix A Table A3) for 15 low-chill and 12 high-chill peach cultivars. The accuracy of the PCA for most markers was higher than 80%, including 14 markers that showed 100% agreement with the results of the Sanger sequencing validation. All 22 SNPs were observed to have only two types of sequence variants for each SNP position. Notably, the presence of certain SNPs was relatively higher in either the low-chill or high-chill cultivars. For example, SNP_IGA_122351, a selected SNP marker linked to major-effect CR-related QTLs, was homozygous for the A/A genotype in 12 out of 12 high-chill cultivars, and this SNP presented the G/G or A/G genotypes in 14 out of 15 low-chill cultivars (it presented the A/A genotype in the low-chill ‘Kuu Taur’ cultivar), suggesting that A/A is associated a high-chill genotype (Table 3). Another major-effect SNP, SNP_IGA_427604, was homozygous for the G/G genotype in 15 out of 15 low-chill cultivars but homozygous for only 2 out of 11 high-chill cultivars, suggesting a low-chill associated role of the G/G genotype (Table 3). To further support the association between genetic markers and CR traits, a chi-squared (*X^2^*) goodness-of-fit test was used to assess the observed versus expected genotype frequency in low-chill and high-chill peach cultivars. All 11 major-effect markers were significantly associated (*p* < 0.05) with CR traits, and seven of them presented *p* values < 0.001 (Table 3). Taken together, the developed PCA pipeline for HRM analysis was successfully applied with 22 CR-related markers for the genotyping of 27 peach cultivars, and potential low-chill associated genotypes for these SNPs were observed.

### 2.5. Potential CR-Related Haplotypes

**Figure 3:**
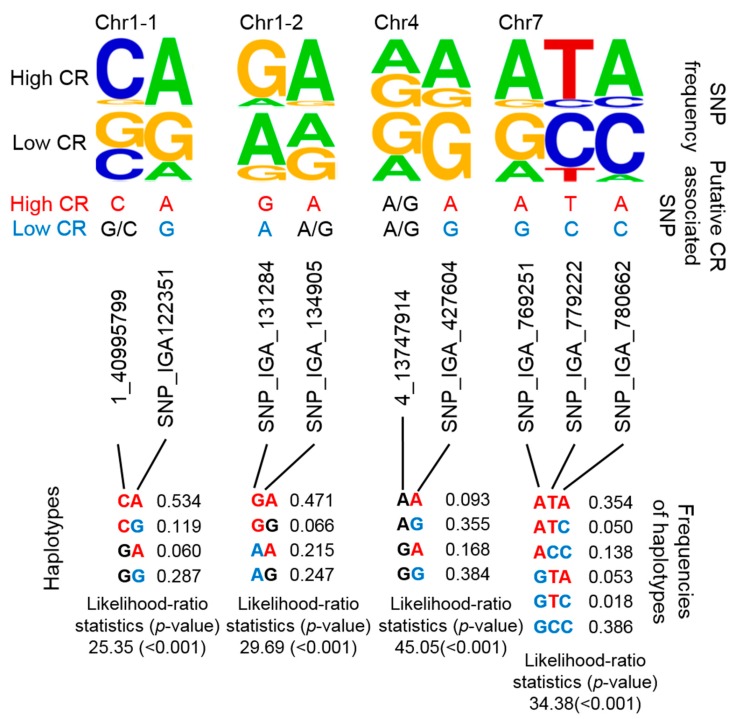
Summary of 4 potential CR-related marker sets based on putative haplotyping. The SNP frequency is reflected by the stack symbol height (upper panel) generated by WebLogo [45]. Putative CR-associated SNPs for high CR (red text) or low CR (blue text) is based on SNP frequency and association analysis. CHAPLIN software was used for haplotype analysis and to determine haplotype frequencies.

By combining several SNPs at a specific locus into a single haplotype, the haplotype would then represent multiple CR-related QTLs. Although it would have been a more stringent to develop haplotypes that encompassed each previously reported QTL, we alternatively considered that nearby regions were putative haplotypes given that CR-related QTLs were mainly located on nearby regions of chromosome (Chr) 1, 4, and 7 (Figure 1). Instead of determining the genotypes of multiple CR-related SNPs, we designed four putative haplotypes to represent these four CR-related loci. Two, 2, 2, and 3 SNPs were used in the haplotype analysis of CR-related loci on Chr 1 (i.e., two positions: Chr1-1 and Chr1-2), Chr 4, and Chr 7, respectively (Figure 3). The frequencies of certain genotypes in some of these SNPs were enriched in high-CR or low-CR cultivars (Figure 3, upper panel). Haplotypes were inferred using the Expectation-Conditional-Maximization (ECM) algorithm (CHAPLIN software) [43,44]. For Chr1-1 and Chr1-2, the frequencies of the CA haplotype (53.4%) of Chr1-1 and the GA haplotype (47.1%) of Chr1-2were high and may represent high CR traits (Figure 3, bottom panel, left). The AA and GA haplotypes of Chr4 could also represent high CR traits (Figure 3, bottom panel, middle). Furthermore, the ATA haplotype of Chr7 may represent high CR traits, while GCC and ACC haplotypes could represent low CR traits (Figure 3, bottom panel, right).

## 3. Discussion

### 3.1. Breeder Toolbox for Peach CRs

In agreement with qualitative trait approaches, we developed a toolkit in this study to analyze markers associated with a quantitative trait, CR, based on the allelic positions from QTL studies for the further implementation of MAS. Prior to this study, breeders would need to retrieve the genomic sequences close to the CR-related markers from the breeder’s toolbox marker converter of the Genome Database for Rosacea [40]. To determine the variants of the retrieved markers, intensive work and validation are required, including the design of primers or probes, the validation of primer efficiency and specificity, the collection of germplasms with low and high CR, and the validation of variant calls using Sanger sequencing. Our study presents a comprehensive approach with experiment-based primer validation that was successfully applied to a collection of peach cultivars with low and high CR levels. A free and open-source R script for HRM variant calling is provided with step-by-step user instructions in addition to a data input template (Supplementary Materials). Alternatively, SNP markers for peach CR-related QTLs are available from previous studies [8,9,10], which can be detected using either GBS or SNP arrays if the breeder is able to afford the expense, equipment, and possesses the knowledge and facilities required for an integrated analysis. In addition, targeted genotyping and sequencing services are available from commercial companies, such as SeqSNP by LGC Biosearch Technologies (Petaluma, CA, USA). As such, indicating the SNPs of interest provided in this study to these services could be an efficient evaluation approach.

### 3.2. Advantages and Limits of Using HRM for Genotyping

Although GBS and SNP arrays are high-throughput approaches to determine the genotypes of the markers linked to the CR-related QTLs [8,9,10], some main issues impeding their widespread use in common breeding operations have not yet been resolved. First, no low-cost and high-efficiency genotyping method has been proposed for the genotyping of peach CR-associated SNP markers prior to this study. Either GBS or the peach 9 K Infinium II array may not be cost-effective for the genotyping of only 10–20 markers with hundreds of samples. Several CR-related QTLs can also be genotyped by SSR markers [8], which may be cost-effective as this method only requires the use of common lab equipment. Nevertheless, there are some drawbacks associated with the use of SSR markers. For example, when assessing the genotypes of the ‘Hongqingshui’ cultivar, the amplification efficiency for ssrPACITA21 was observed to be very low, and multiple products were found in the PCRs of AMPA103 and PacC13, indicating that the PCR amplification efficiency and specificity of some of the SSR primer pairs were low for some cultivars. Additionally, genotyping using the gel-based SSR method is relatively labor-intensive. This limits the SSR method from being applied in large-scale genotyping projects and makes it particularly susceptible to human error.

We propose that HRM may be a more suitable method for the genotyping of CR-related markers. Firstly, HRM instruments and reagents are less expensive and more universal than those of either the microarray or sequencing approaches. Secondly, the in silico genotyping pipeline provided here not only increases the throughput but also decreases the chance of producing biased results due to human error. Although the singleplex nature of HRM limits its throughput potential for detecting large numbers of SNPs, HRM remains a rapid and simple solution for genotyping peach CR-related markers when compared to that of GBS or SNP arrays. Among all 22 selected SNPs representing the CR-related QTLs, 11 of the selected SNPs were linked with major-effect cofactors (Figure 1), which are the first priority for the implementation of MAS. With respect to qualitative traits, the SNPs associated with six qualitative traits were identified [37,39]. If one SNP represents each qualitative trait and the HRM-PCA pipeline is adopted, a breeder toolbox consisting of a total of 17 SNPs associated with quantitative CR traits and six qualitative traits could be developed in the future. HRM-based methods would thus represent an efficient approach for analyzing this number of markers.

### 3.3. Advantages of Using PCA for Variant Calls

To date, commercialized software packages, such as HRM Software v. 2.0 (Applied Biosystems, Waltham, MA, USA), are probably still the most common tools for analyzing HRM results, but there are some drawbacks associated with the use of these commercial tools. In this study, when HRM Software v. 2.0 (Applied Biosystems) dealt with the widespread melt curves, its accuracy decreased. For example, the accuracy of HRM Software v. 2.0 (Applied Biosystems) for analyzing SNP_IGA780662, SNP_IGA786935, and 8_17718144 was only 55.6%, 64.3%, and 80%, respectively (Figure 2). In addition, the price of commercial software may represent a huge expense for some breeders with limited budgets. An alternative statistical software, ScreenClust (Qiagen, Venlo, The Netherlands), has been developed to improve the HRM allele assortment and is powered by PCA clustering [27]. By adopting this concept and simplifying the analysis approach, we developed an R script using PCA for melt curve clustering. As a result, our R script presents several advantages. First, the accuracy of our R script is higher when compared to that of HRM software v. 2.0 (Applied Biosystems). For example, the accuracy of our R script for analyzing SNP_IGA780662, SNP_IGA786935, and 8_17718144 was 100%, 92.9%, and 100%, respectively (Figure 2). Moreover, our R script is free and can be modified by users according to their needs. Not only are step-by-step user instructions described in the Materials and Methods section, but the R script and an input data template file are also available in the Supplementary Materials.

### 3.4. Putative Low CR-Assoicated SNPs Are Potential Candidates for MAS

When reviewing most of the selected markers in the cultivar collection, the frequency of one specific SNP variant was found to be much higher in cultivars with low CR than that of cultivars with high CR (Table 3). These types of SNPs are considered to be putative low-chill associated SNPs. For example, with SNP_IGA_780662, there are more C/C homozygous genotypes observed in the cultivars with low CR, while more A/A homozygotes or A/C heterozygotes are found in high-chill cultivars. Two popular high-chill cultivars, ‘Hongqingshui’ and ‘Nakatsu Hakuto,’ in the Taiwan market both show the A/A genotype at this SNP site. The association between genotypes and CR traits is strengthen by the results of our chi-squared test, and this SNP site was found to be highly significant (*p* < 0.001). Although more germplasms and SNPs in the genome may be required to further the statistical support for this association, it is still very likely that the C/C genotype is a low-chill specific SNP for SNP_IGA_780662 (Table 3). The putative low chill-associated SNPs of most of the selected markers have been identified (Table 3 and Appendix A Table A3), and they could be potential genotypes for the selection of parental germplasms and candidate progenies for breeding cultivars with low CR.

### 3.5. Recommended Marker Lists for Users of This Toolkit

When linkage maps and QTLs are built for traits controlling the CR [7,8,9,10], it becomes possible to carry out CR MAS at the seedling stage. Three peach F_2_ populations have been employed for the mapping of these QTLs [7,8,9,10]. The molecular markers used for genotyping the first F_2_ population were SSRs and amplified fragment length polymorphism (AFLP) markers [7]. This was followed by re-sequencing to refine the QTL resolution and develop gene-targeted SSR markers [8]. The other two F_2_ peach populations have been mapped using high-throughput SNP arrays and GBS [9,10]. To adopt such various marker systems for MAS, SNP arrays or GBS have been considered to be useful tools to integrate the markers from the three QTL maps, but these tools require advanced facilities and bioinformatic analysis, which represent a barrier to breeders from common labs, as previously discussed. Another feasible strategy is to adopt only one of these three QTL maps and stick with the original marker system used for mapping. By doing so, breeders can avoid the processes of QTL integration and the installation of multiple marker systems in their labs. Nevertheless, a main issue related with the use of only one QTL map derived from one F_2_ population is the lack of information of other major-effect QTLs mapped by other F_2_ populations. This issue was highlighted in the integration of information in this study given that many major-effect SNP markers that originated from different maps did not overlap and were not co-localized on the physical map produced (Figure 1). To address these issues, this study has contributed a simplified and cost-effective HRM-PCA pipeline, integrated with most of the available major-effect CR-related markers and with a low entry threshold for peach breeders. To further reduce the costs and labor associated with genotyping all selected markers, we offer recommendations to prioritize candidate markers.

The nature of a quantitative trait, such as the CR, is that the trait is controlled by multiple genes and some of these genes contribute to a higher genetic variance of the trait. These genes are known as major-effect genes. As such, selecting genes with high effectiveness and gene pyramiding are key concepts for the prioritization of candidate markers. To this end, markers in this study were categorized as either major- or minor-effect markers according to their contribution to the CR (Figure 1; Table 1; and Appendix A Table A1). Among these markers, 11 SNPs for major-effect QTLs are recommended to breeders as part of a priority list for genotyping. Considering the concept of gene pyramiding, these markers have been suggested so that breeders may survey the genotypes of their germplasm collections.

With respect to the technical limitations of the HRM-PCA method and the polymorphic diversity of the CR-related markers in the peach gene pool, the marker lists provided in this study may even be shortened depending on the accuracy of the HRM primer pairs and the associations between markers and traits. Even though we have optimized the amplification efficiency for all primer pairs to nearly 100%, some of the primer pairs still exhibit lower accuracies than the others (Table 3 and Appendix A, Table A3). For example, the accuracy of SNP_IGA_134905, 1_40995799, and 4_13747914 was 66.7%, 85.7%, and 80%, respectively. For breeders with limited budgets, we recommend only using markers with high accuracy for major-effect QTLs. Similarly, for breeders who aim to genotype minor-effect QTLs, we recommend first selecting the markers with 100% accuracy. Considering the significant level of association between genetic markers and peach CR traits, a contingency table chi-squared test was carried out to assess this association. A concise list of markers can be generated by selecting the markers with highly significant levels of association. Since 11 major-effect markers and 4 minor-effect markers were found to be significantly associated (*p* < 0.05) with CR-related traits (Table 3 and Appendix A Table A3), the selected markers can be filtered down from 22 to 15 markers according the association level. Alternatively, putative haplotypes on three loci would be useful to simplify the genotyping for peach CR traits (Figure 3, bottom). These three sets of putative haplotypes can be assessed by determining a total of only 9 SNPs. Nevertheless, this haplotype analysis was based on only 27 germplasms that are relevant to CR studies of peach cultivars in the Taiwan and Southeast Asia regions, so a large number of progeny or peach germplasms that have already been phenotyped for the CR may be required to confirm the association of these putative haplotypes.

In summary, although a total of 22 markers are provided in this study, we recommend that breeders use the marker sets that represent putative haplotypes. If possible, breeders can also select several or all of the markers that are linked with major-effect and/or minor-effect QTLs. We suggest that breeders adjust the number of markers by considering the accuracy of the HRM primer pairs and the significance of the associations between markers and traits.

## 4. Conclusions

This study provides a toolkit with optimized primers and experimental settings for assessing the genotypes of 22 SNP CR-related peach markers, including 11 markers linked to major-effect QTLs and another 11 markers linked to minor-effect QTLs. A cost-effective HRM genotyping system was connected with a free and open-sourced R-based PCA variant calling pipeline to empower this toolkit with high accessibility and flexibility for peach breeders. This pipeline has successfully determined genotypes for a collection of peach germplasms consisting of low-chill and high-chill cultivars. Although SNP arrays and GBS are high-throughput genotyping approaches, this simplified and rapid toolkit still has high implementation potential. With this toolkit, breeders are able to assess the genotypes of germplasms, screen a large number of progeny during the early developmental stages, and accelerate peach breeding using MAS for quantitative CR traits. A future extension of this HRM-based toolkit is slated to include SNPs and putative haplotypes linked with qualitative peach traits, which will further establish this toolbox as a comprehensive tool for determining quantitative CR traits and multiple qualitative traits. Considering the germplasms evaluated in this study, the recommended markers are applicable for the germplasms relevant to the Taiwan and Southeast Asia regions, and further studies are needed to validate these SNPs as CR markers in other germplasms outside of Taiwan and Southeast Asia.

## 5. Materials and Methods

### 5.1. Plant Materials

The young and fully expanded leaves of a total of 27 peach (*Prunus persica* L.) cultivars, spanning low to high CRs, were collected for HRM genotyping. The CR phenotypes (i.e., chilling hours) of 20 cultivars have been previously reported (Table 3) [46,47,48,49,50,51,52,53,54] and were used for association analysis. Fifteen peach cultivars including ‘Flordabeauty’, ‘Okinawa’, ‘Premier’, ‘Xiami’, ‘Tropicprince’, ‘Kuu Taur’, ‘Yinggetao’, ‘Flordared’, ‘Chuenfeng’, ‘SpringHoney’, ‘Tropicsweet’, ‘Ruby’, ‘Tropicsnow’, ‘Fushou’, and ‘Flordabelle’ were sampled from the Taiwan Agricultural Research Institute in Taichung, Taiwan. The other 12 peach cultivars including ‘Hongqingshui’, ‘Nakatsu Hakuto’, ‘Sunago wase’, ‘Okubo’, ‘Yamato Wase’, ‘Yamane Hakuto’, ‘Okitsu’, ‘Tsao Sheng Yu Tao’, ‘Shanghaishuimi’, ‘Aki Hakuto’, ‘Odama Hakuho’, and ‘Shiga Hakuto’ were collected from the Highland Experimental Farm of the National Taiwan University in Nantou County, Taiwan. All samples were stored at –80 °C prior to DNA extraction. These cultivars were mainly selected based on the availability of CR records and by considering both their flavor and adaptation for East Asia and Southern East Asia, especially Taiwan.

### 5.2. Genomic DNA Extraction

Genomic DNA was extracted from peach leaf tissues by a modified cetyltrimethyl ammonium bromide (CTAB) method [55]. Briefly, peach leaves were homogenized using a pestle and mortar in liquid nitrogen. One gram of the homogenized sample was resuspended with 11 mL of DNA extraction buffer (100 mM Tris-HCl at pH 8.0, 25 mM EDTA, 1.4 M NaCl, 0.1% polyvinylpyrrolidone with an average mol wt of 40,000 (PVP-40), 2% CTAB, 0.2% β-mercaptoethanol, and 0.15 mg·mL^−1^ Proteinase K). After being mixed well via inversion and incubated at 65 °C for 30 min, the extract was centrifuged at 3000× *g* for 10 min. The supernatant was transferred to a fresh tube and then mixed with the same volume of chloroform:isoamyl alcohol (24:1), followed by centrifugation at 8000× *g* for 10 min. The upper aqueous phase was transferred to a new tube, and a half volume of 5 M NaCl and a 0.6–0.7 volume of ice-cold isopropanol were added. After standing the mixture at 25 °C for 1 h, precipitated genomic DNA was pelleted by centrifugation at 10,000× *g* for 20 min. The genomic DNA pellet was washed with 70% ethanol and air dried. The dried pellet was dissolved in 0.5 mL of high salt TE buffer (1 M NaCl, 10 mM Tris-HCl pH 8.0, and 1 mM EDTA). To clean RNA contamination, the genomic DNA solution was digested by the addition of 0.5 µL RNase A (20 mg·mL^−1^), followed by incubation at 37 °C for 30 min. The RNase-treated DNA was cleaned up again by a chloroform:isoamyl alcohol (24:1) extraction and subsequent alcohol precipitation with a 0.6–0.7 volume of cold isopropanol. After spinning at 12,000× *g* for 30 min at 4 °C, the pellet was washed with 70% ethanol and air dried. The dried genomic DNA pellet was dissolved in DNase-free water for subsequent analysis.

The integrity of the genomic DNA was visualized with 0.8% agarose gel electrophoresis. The DNA concentration and purity (A_260_/A_280_ and A_260_/A_230_ ratios) were evaluated using an Epoch Microplate Spectrophotometer (BioTek, Winooski, VT, USA). The final concentration of DNA was adjusted to 20 ng·µL^−1^ and stored at −20 °C for subsequent HRM analysis.

### 5.3. HRM Analysis

Specific primers with a near−100% PCR efficiency (Appendix A Table A2) were designed for the HRM assays. To increase PCR efficiency, primer pairs are designed for amplicons with lengths shorter than 150 bp [56]. In addition, PCR efficiency was determined by the construction of optimal standard curves with Ct values and genomic DNA quantity. Briefly, peach leaf genomic DNA was serially diluted with deionized water and used as a template for qPCR analysis. The PCR efficiency was calculated with StepOnePlus v. 2.3 software (Applied Biosystems, Waltham, MA, USA) using a formula of PCR efficiency (PCR efficiency = 10^−1^/slope^−1^) with two technical repeats. HRM was conducted with 2.5 ng of genomic DNA as a template using a StepOnePlus Real-Time PCR System (Applied Biosystems) and MeltDoctor HRM Master Mix (Applied Biosystems) according to the instructions from the manufacturer. The HRM thermocycles were set as follows: enzyme activation at 95 °C for 10 min, 40 cycles of denaturation at 95 °C for 15 s, and annealing/extension at 60 °C for 1 min. Prior to the melting curve analysis, the PCR amplicons were denatured at 95 °C for 15 s and reannealed at 60 °C for 1 min. The melting curves were generated by dissociation at 95 °C with a 1% ramp rate. The amplification and dissociation curves were analyzed using StepOnePlus v. 2.3 software (Applied Biosystems). In addition, a melt curve analysis at the post-PCR step was carried out using HRM software v. 2.0.1 (Applied Biosystems).

### 5.4. PCA for HRM Output Result Clustering 

After HRM analysis, raw melt region temperature data and melt region normalized fluorescence data were exported with the StepOnePlus v. 2.3 software. Normalization and PCA were performed on the HRM output raw fluorescence data in RStudio [42]. The samples were clustered and visualized using the ‘mclust’ [57] and ‘plot3D’ packages, respectively. To verify the clustering results of the HRM data analyzed via PCA, randomly selected samples from each cluster were sequenced by Sanger sequencing (performed by Mission Biotech Co., Taipei, Taiwan). All R studio scripts are available (Supplementary Materials, also available on GitHub, https://github.com/choulin2/PCA_HRM.git). Please see Section 5.5 for step-by-step PCA pipeline instructions.

### 5.5. Instructions of the HRM Fast Genotyping Platform Analyzed With the PCA Pipeline

After PCR amplification and HRM dissociation, temperature data and normalized fluorescence data of the melt region may be exported as a single file with StepOnePlus v. 2.3 software (Applied Biosystems, Waltham, MA, USA). Afterwards, the temperatures of all wells on each fluorescence read are averaged to represent the temperature of each fluorescence read. All single SNP fluorescence data are listed with the corresponding temperature on each read. This data should be arranged based on the format of the provided template (please see Supplementary Materials “PCA_HRM.example.csv”, also available on GitHub, https://github.com/choulin2/PCA_HRM.git) and saved as a ‘.csv’ file using Microsoft Excel (Microsoft, Redmond, WA, USA).

After converting the raw data into this R-readable format, the R scripts we provided (see Supplementary Materials “PCA_HRM.v8.R”, also available on GitHub, https://github.com/choulin2/PCA_HRM.git) can be loaded into R studio. Before running the R scripts, we recommend setting the working directory to the folder containing this R script file and the data .csv file (Session /Set Working Directory /Choose Directory). Then, the data can be imported by running the following scripts:

> input_file = readline(‘Enter the file name: ’)
> exported.data.file = read.csv(input_file,header = T)

After data input is complete, the upper and lower melt temperature limits may be set according to the optimized temperature for each marker (Table 2) as follows:

> max_lim = readline(‘Enter the upper limit of the melt region: ’)
> min_lim = readline(‘Enter the lower limit of the melt region: ’)

After setting the melt region for analysis, the scripts may be directly run line-by-line from step 2 to step 6 to complete data normalization and conduct the PCA. Afterwards, the latest version of the ‘mclust’ and ‘plot3D’ packages should be installed from The Comprehensive R Archive Network (CRAN, https://cran.r-project.org/) for clustering and three-dimensional (3D) graph plotting:

> library(mclust)
> library(plot3D)

Two or three principal components (PCs) were chosen for clustering at step 7 based on the explained variance of each principal component (PC) after the PCA:

> cluster.data <- Mclust(PCA.analysis.file$rotation[,1:3], G = 3)
> df.PCA.MM1 <- as.data.frame(PCA.analysis.file$rotation[,1:3])
> cluster.data$classification

In the above script, both of the selected PCs and the expected genotype number for clustering are adjustable. The number of selected PCs may be adjusted by changing the number of analyzed columns from PCA.analysis.file$rotation[,1:3], while G defines how many expected genotype groups were used for clustering.

In order to visualize the clustering results, scatter diagrams of the chosen PCs may be plotted with the ‘plot’ function of step 7. In addition, the ‘identify’ function was used to identify selected points on the interactive graphs by pressing the mouse button over the desired point.

> plot(df.PCA.MM1[,1:2], bg=cluster.data$classification, pch=21,xlab='PC1', ylab='PC2')
> identify(df.PCA.MM1[,1:2], labels = rownames(df.PCA.MM1))

After running the ‘identify’ function, it is necessary to press the ‘esc’ button or the finish tab in order to finish the interactive plot and to run the following scripts. In the last data export step, 3D clustering figures may be obtained, such as in Figure 2, in ‘.pdf’ format as well as a variant calling file, such as “PCA_HRM.example.csv variant call.csv” (see Supplementary Materials). According to the information of the variant call, samples that are clustered in the same group may be identified. A web-based demonstration version of this R script and example data can be accessed on NextJournal (https://nextjournal.com/RNA-Sick/hrm-analysis-with-pca-and-clustering). To validate genotypes, at least one selected sample from each group is subjected to Sanger sequencing, and the represented genotype of each variant call may be defined. In this manner, we may genotype all samples *in silico* for all the selected CR-related markers (Table 3 and Appendix A Table A3).

### 5.6. Association Analysis and Haplotype Analysis

Peach cultivars were classified by high and low CR, and SNP markers were grouped by genotypes. An association analysis between genetic markers and peach CR traits was performed using a chi-square test in a contingency table that contained the counts of individuals in each category. The chi-squared test statistic and *p* value of the test were calculated with the ‘CHITEST’ function in Microsoft Excel to assess the changes of the observed versus expected counts in each category. Regarding the haplotype analysis, genotype data of SNPs in three chromosome regions from all cultivars were converted to “0”, “1”, or “2”. The homozygous AA SNP sequence was “0”, the heterozygous AB sequence was “1”, and the homozygous BB sequence was “2”. These genotypic information were then formatted for Case-control haplotype inference (CHAPLIN) software [43,44], setting low CR and high CR as the case and control, respectively. The modeling of this likelihood approach was set as ‘general’.

## Figures and Tables

**Table 1:** Major-effect peach chilling requirement (CR)-related quantitative trait loci (QTLs) and the representative markers.

Selected Marker ^1^	Original Cofactor ^2^	QTL ^3^	LOD ^4^	R^2^ (%) ^5^	Physical Position (Original Cofactor) ^6^	Marker Distance From The Cofactor ^7^
Romeu et al., 2014 [9]						
SNP_IGA_122351	SNP_IGA_122057	CRW-AA/EJ CRU-AA/EJ CRD-AA/EJ	16.3–22.3	64–76	Pp01:41981168	+20228
SNP_IGA_779222	SNP_IGA_779224	CRW-AA CRU-AA/EJ CRD-AA/EJ	3.2–4.5	14–25	Pp07:15717070	−138
SNP_IGA_769251	SNP_IGA_769194	CRW-EJ CRU-EJ	3.7–3.9	18–20	Pp07:12156489	+3271
Zhebentyayeva et al., 2014 [8]						
SNP_IGA_134905	Pchgms29	qCR1a-2008 qCR1a-2009	46.8 (2008) 17.8 (2009)	44.1 (2008) 19.1 (2009)	Pp01:43499388..43499459	372
SNP_IGA_381567	Pchgms174	qCR4a-2008 qCR4a-2009	10.3 (2008) 2.6 (2009)	11.4 (2008) 3.4 (2009)	Pp04:2431831..2431882	−2207
SNP_IGA_780662	UDAp-460	qCR7-2009	2.4	24.4	Pp07:16270932..16270953	+2102
SNP_IGA_786935	UDAp-409A	qCR7-2008	17.2	18.5	Pp07:19150241..19150290	−9274
Bielenberg et al., 2015 [10]						
1_40995799	1_40995799	qCR1-2009	4.62	24.8	Pp01:41969066	N/A ^8^
SNP_IGA_131284	1_44762763	qCR1-2008	12.78	16.0	Pp01:45053074	4078
SNP_IGA_427604	4_14984691	qCR4-2009	5.13	27.8	Pp04:14995235	−220
4_13747914	4_13747914	qCR4c-2008	12.29	14.9	Pp04:13758549	N/A

^1^ Selected marker: the flanking single nucleotide polymorphism (SNP) marker representing the original cofactor as the selected SNP marker. ^2^ The original cofactor linked with QTL. ^3^ QTL: quantitative trait loci identified in each F_2_ population. ^4^ LOD: logarithm of the odds. ^5^ R^2^ (%): percentage of phenotypic variance explained by the QTL. ^6^ Physical position: the position of the original cofactor on the *P. persica* genome v.2.0.a1 physical map. The physical position of each marker was defined by scaffold and bp position (scaffold:bp position for SNP and scaffold:bp.bp region for simple sequence repeat (SSR)) in the *P. persica* genome v.2.0.a1^7^ Marker distance from the cofactor indicates the upstream (+) or downstream (−) distance of the cofactor from the selected SNP marker. When the cofactor is an SSR marker, the distance is counted from the side closest to the selected SNP marker (i.e., from the left side of the SSR for upstream SNPs; from the right side of the SSR for downstream SNPs). ^8^ N/A: not applicable.

**Table 2:** Primer pairs and parameters for the high resolution melting (HRM) analysis of the SNP markers.

		Optimized Temperature Regions for Normalization (°C)			
Selected Marker ^1^	Primer Pairs	Applied Biosystems HRM Software v2.0		Principal Components Analysis	
		Pre-Melt Region ^2^	Post-Melt Region ^2^	Lower Limit ^3^	Upper Limit ^3^
SNP_IGA_122351	S1_4102b-f1/r1	72.6–73.0	82.0–82.5	73.0	82.0
SNP_IGA_297497	S3_0363-f2/r2	69.1–69.5	79.8–80.2	63.5	79.0
SNP_IGA_769251	S7_1256-f1/r1	78.7–79.1	87.0–87.5	79.1	87.0
SNP_IGA_779222	S7_1611-f1/r1	72.7–73.1	81.8–82.3	77.5	82.0
rs159238319	S1_1690-f2/r2	67.1–67.5	77.1–77.6	67.5	77.0
SNP_IGA_134905	S1_4631-f2/r2	74.5–74.9	82.6–83.1	73.0	82.5
SNP_IGA_381567	S4_2429-f2/r2	68.7–69.1	78.1–78.6	69.0	78.0
SNP_IGA_419106	S4_1351-f1/r2	69.6–70.1	76.3–76.8	70.1	76.3
SNP_IGA_695463	S6_2645-f2/r2	73.6–74.0	83.2–83.7	74.0	83.5
SNP_IGA_786935	S7_1954b-f1/r1	77.2–77.6	83.9–84.4	77.6	83.9
SNP_IGA_112592	S1_3674-f2/r2	68.6–69.0	77.8–78.2	69.0	78.5
rs159239801	S4_9208b-f1/r1	71.0–71.5	77.2–77.7	71.5	77.7
SNP_IGA_780662	S7_1667-f1/r1	75.7–76.0	85.2–85.6	77.5	85.0
SNP_IGA_131284	S1_4475-f1/r1	71.4–71.8	79.5–80.0	71.8	79.5
1_40995799	S1_4099-f1/r1	74.2–74.4	82.1–82.5	74.0	82.5
2_16900230	S2_1690-f1/r1	79.1–79.3	85.5–86.2	79.3	85.5
4_00772820	S4_0077-f1/r1	72.1–72.3	80.2–80.6	72.5	80.0
4_11060745	S4_1106-f1/r1	77.9–78.1	85.4–85.8	77.0	85.5
4_13747914	S4_1374-f1/r1	74.8–75.0	81.1–81.3	75.0	81.0
SNP_IGA_427604	S4_1498b-f2/r2	72.9–73.3	80.5–81.0	73.0	81.0
5_13713689	S5_1371-f2/r2	74.7–75.2	82.5–83.0	75.0	82.5
8_11718744	S8_1171-f1/r1	74.2–74.6	80.9–81.5	70.5	80.5

^1^ Selected marker: the flanking SNP marker replacing the original cofactor as the selection marker. The related cofactors are shown in Table 1 and Appendix A Table A1. ^2^ Pre-melt/ Post-melt region: the temperature regions before/after the active melt region that are used to align the data and perform clustering with the Applied Biosystems High Resolution Melt software v. 2.0. ^3^ Lower / Upper limit: the minimum/maximum temperatures of the active melt region that are used to align the data and perform clustering via PCA in R studio.

**Table 3:** Genotypes of the selected SNP markers linked to major-effect CR-related QTLs in 27 peach cultivars.

Cultivar ^1^	CR (h)	SNP_IGA_122351	SNP_IGA_769251	SNP_IGA_779222	SNP_IGA_134905	SNP_IGA_381567	SNP_IGA_786935	SNP_IGA_780662	SNP_IGA_131284	1_40995799	4_13747914	SNP_IGA_427604
Low-chill cultivars												
Okinawa	100	G	G^†^	C	A	G	A^†^	C^†^	G	C	G	G
Flordared	100	G	A/G	C	G	A/G	A^†^	C	A^†^	G	A/G	G^†^
Ruby	100	A/G	G	C^†^	A/G^†^	A/G^†^	A/G^†^	C	A/G	C/G^†^	G	G
Xiami	125	G	A/G	C	A	A/G	A^†^	C	A^†^	C	A/G^†^	G
Yinggetao	125	G^†^	G	C	G^†^	G	A^†^	C	G	G^†^	G	G
Premier	150	G	A/G	C	A	A/G	A^†^	C	A	C	A/G^†^	G
Flordabelle	150	A/G^†^	A/G	T/C	G	G^†^	A	C	A	C/G^†^	G^†^	G
Flordabeauty	150	A/G^†^	G^†^	C^†^	A	A	A/G^†^	C	A	C/G^†^	A/G	G
TropicPrince	150	A/G	G^†^	C	G	A	G^†^	C^†^	A	G	A^†^	G
Kuu Taur	150	A	A^†^	T	A^†^	G	A^†^	C	A	C^†^	G^†^	G
Chuenfeng	150	G	A/G	C	G	A^†^	A	C^†^	A	G	A/G^†^	G
TropicSweet	175	A/G	G	T/C^†^	G	A/G	A/G	C/A	A^†^	C/G	A/G	G
SpringHoney	180	G	G	C	A/G	A/G	A/G^†^	C	A/G^†^	G	A/G	G
Tropicsnow	200	A/G	A/G	T/C^†^	A	G^†^	A/G^†^	C/A	A	C	A/G	G
Fushou	n.a. ^5^	G	A/G	T/C^†^	A/G	G	A	C/A^†^	A/G^†^	G	A/G^†^	G^†^
High-chill cultivars												
Yamane Hakuto	800	A	A	T	A^†^	A	G	A	G	C	G	A
Shiga Hakuto	800	A^†^	A	T^†^	A^†^	A	G	A^†^	G^†^	G	A^†^	A/G^†^
Okubo	850	A	A	T	A	A	G	A	G	C	G	A
Shanghaishuimi	850	A	A/G^†^	T	A	A	A/G^†^	C/A^†^	A/G^†^	C	A/G	A/G^†^
Okitsu	900	A	A	T/C	A	A	A	C/A^†^	G^†^	C	A	G
Aki Hakuto	900	A	A^†^	T	A^†^	A	G^†^	A	A/G	C	G	A
Hongqingshui	N/A	A	A	T	A/G^†^	A	G	A^†^	A/G	C^†^	A^†^	A
Nakatsu Hakuto	N/A	A	A^†^	T^†^	A^†^	A	G	A	G	C	G	A^†^
Sunago wase	N/A	A	A/G^†^	T	A	A/G	A/G	A	G	C	A/G	A/G^†^
Yamato Wase	N/A	A^†^	A	T	A/G^†^	A	G	A^†^	G	C^†^	A	A^†^
Odama Hakuho	N/A	A	A	T^†^	A	A	G^†^	A	G^†^	C	G	A^†^
Tsao Sheng Yu Tao	N/A	A	A/G^†^	C^†^	A	A^†^	A/G	C	G	C	A^†^	G^†^
Putative low chill associated marker		G	G	C	G	G	A	C	A	G	G	G
Significance (*X^2^*-test) ^3^		***	***	***	*	***	**	***	***	**	*	***
Accuracy (ratio; %) ^4^		9/9; 100%	9/9; 100%	9/9; 100%	6/9; 66.7%	8/8; 100%	13/14; 92.9%	9/9; 100%	9/9; 100%	6/7; 85.7%	8/10; 80%	9/9; 100%

^1^ Cultivar name: for cultivars without English names, the names were transliterated based on the pronunciation of their Japanese or Chinese name. ^2^ Putative low chill specific markers: the SNP sequence variant is relatively more frequent in low-chill cultivars than in high-chill cultivars. ^3^ Association analysis between genetic markers and peach chilling requirement (CR) traits using a contingency table chi-squared (*X^2^*) test. * *p* < 0.05, ** *p* < 0.01, and *** *p* < 0.001. ^4^ The accuracy of each marker was calculated as follows: the number of variant calls consistent with Sanger sequencing / total sample number sequenced with Sanger sequencing in both the ratio and percentage. ^5^ N/A: not available. ^†^ The genotyping results validated with the Sanger sequencing method.

## Supplementary Materials

Supplementary materials can be found at https://www.mdpi.com/1422-0067/21/4/1543/s1.

## Abbreviations

HRM	High resolution melting analysis
CR	Chilling requirement
QTL	Quantitative trait locus
PCA	Principal component analysis
MAS	Marker-assisted selection
SSR	Simple sequence repeat
SNP	Single nucleotide polymorphism
GBS	Genotyping-by-sequencing
PCR	Polymerase chain reaction
KASP	Kompetitive allele-specific PCR
FRET	Fluorescence resonance energy transfer
qPCR	Quantitative PCR
LOD	Logarithm of the odds
LG	Linkage group
X^2^	Chi-square
EndoPGs	Endopolygalacturonases
NBS-LRR	Nucleotide binding site leucine-rich repeat
AFLP	Amplified fragment length polymorphism
CTAB	Cetyltrimethyl ammonium bromide
PVP-40	Polyvinylpyrrolidone average mol wt 40,000
CRAN	The Comprehensive R Archive Network
3D	Three-dimension
PC	Principal component

## Appendix A

**Table A1:** Minor-effect peach chilling requirement (CR)-related quantitative trait loci (QTLs) and the representative markers.

Selected Marker ^1^	Original Cofactor ^2^	QTL ^3^	LOD ^4^	R^2^ (%) ^5^	Physical Position (Original Cofactor) ^6^	Marker Distance from the Cofactor ^7^
Romeu et al., 2014 [9]						
SNP_IGA_297497	SNP_IGA_297497	CRW-EJ	3.8	10	Pp03:3635392	N/A ^9^
N/A ^8^	SNP_IGA_293752	CRD-EJ	2.5	6	Pp03:1985533	
N/A	SNP_IGA_635355	CRU-EJ CRD-EJ	2.8-3.8	13-18	Pp06:10237718	
Zhebentyayeva et al., 2014 [8]						
rs159238319	UDA-053	qCR1d-2008 qCR1d-2009	2.9 (2008) 6.7 (2009)	3.1 (2008) 6.9 (2009)	Pp01:1689830..1689847	+119
SNP_IGA_112592	BPPCT036B	qCR1c-2009	2.4	2.7	Pp01:37719340..37719361	+55
N/A	Pchgms170	qCR2-2009	2.3	3.4	Pp02:16917187..16917222	
SNP_IGA_419106	AMPA103	qCR4b-2008	3.0	4.6	Pp04:13519992..13520025	+7012
rs159239801	M12a	qCR4b-2009	2.7	3.6	Pp04:9219635..9219660	−264
N/A	ssrPACITA21	qCR5-2008 qCR5-2009	3.9 (2008) 3.9 (2009)	4.6 (2008) 4.5 (2009)	Pp05:10776287..10776338	
SNP_IGA_695463	EPPISF002	qCR6-2008	3.4	3.9	Pp06:28325489..28325504	−2022
N/A	PacC13	qCR8-2008 qCR8-2009	4.0 (2008) 2.4 (2009)	5.0 (2008) 2.8 (2009)	Pp08:18135194..18135213	
Bielenberg et al., 2015 [10]						
2_16900230	2_16900230	qCR2-2008	7.72	10.5	Pp02:20476740	N/A
4_00772820	4_00772820	qCR4a-2008	6.23	5.9	Pp04:772922	N/A
4_11060745	4_11060745	qCR4b-2008	5.06	4.5	Pp04:11071616	N/A
5_13713689	5_13713689	qCR5a-2008	6.00	5.7	Pp05:13708460	N/A
N/A	BPPCT038	qCR5b-2008	4.50	4.0	Pp05:14652958..14653005	
8_11718744	8_11718744	qCR8-2008	8.64	9.0	Pp08:12463247	N/A

**^1^** Selected marker: the flanking SNP marker representing the original cofactor as the selected SNP marker. ^2^ The original cofactor linked with QTL. ^3^ QTL: quantitative trait loci identified in each F_2_ population. ^4^ LOD: logarithm of the odds. ^5^ R^2^ (%): percentage of phenotypic variance explained by the QTL. ^6^ Physical position: the position of the original cofactor on the *P. persica* genome v.2.0.a1 physical map. The physical position of each marker was defined by scaffold and bp position (scaffold:bp position for SNP and scaffold:bp..bp region for SSR) in the *P. persica* genome v.2.0.a1. ^7^ Marker distance from the cofactor indicates the upstream (+) or downstream (-) distance of the cofactor from the selected SNP marker. When the cofactor is an SSR marker, the distance is counted from the side closest to the selected SNP marker (i.e., from the left side of the SSR for upstream SNPs; from the right side of the SSR for downstream SNPs). ^8^ N/A: not available, indicating that no suitable primers with high PCR efficiency or specificity are available or that no polymorphisms were found at adjacent SNPs in all tested peach cultivars. ^9^ N/A: not applicable.

**Table A2:** Primer lists of SNP markers for high resolution melting (HRM) analysis.

Selected Marker ^1^	Original Cofactor ^2^	Primer Name	Sequence (5’-3’)	Ta (°C) ^3^	Product Size (bp)	Efficiency of PCR ^4^	R^2^
SNP_IGA_122351	SNP_IGA_122057	S1_4102b-f1	ATTTTGTATCTGCGTGTGGACGGAG	60.1	152	92.579	0.998
		S1_4102b-r1	TGCGGTAATCTAGGAACTGGAGTCG	59.6			
SNP_IGA_297497	SNP_IGA_297497	S3_0363-f2	AGTGACAAGGAAAGTCTCTCTGAAGGC	58.7	81	98.858	0.994
		S3_0363-r2	CTGGCTCAAACACTCAACCAACTTG	58.8			
SNP_IGA_769251	SNP_IGA_769194	S7_1256-f1	CGCACAGATTCCAACAGAGCCG	61.4	104	95.879	0.997
		S7_1256-r1	GCGACTTTGGTCCACGTTATGCC	61.3			
SNP_IGA_779222	SNP_IGA_779224	S7_1611-f1	GACCGAAGAATATCGACGTTAAGGGTTCTTTG	65.1	98	94.51	0.972
		S7_1611-r1	AAAGTTCATGCAGAAGATACCAGCAGACTC	61.3			
rs159238319	UDA-053	S1_1690-f2	CTCTTGTTGGTTATCTCATTGTTAAGTGATTTGACATG	65	77	122.991	0.967
		S1_1690-r2	CAACCACAAGTCTCACAAAATGCACAC	60.5			
SNP_IGA_134905	Pchgms29	S1_4631-f2	TTCTACCAATATGAAAAAGCTACCTGGGGTT	62.2	88	123.755	0.984
		S1_4631-r2	TGATTACCTCCGAGCTTCTGATAGGC	60			
SNP_IGA_381567	Pchgms174	S4_2429-f2	GTGGAGTATCTTCGGAACTCAGAAAACCA	61.8	74	105.302	0.984
		S4_2429-r2	CATGAGATGATCGTCAGTCTAAACTCTTAACTTACC	62			
SNP_IGA_419106	AMPA103	S4_1351-f1	GTGACATTTGACTAGGTCTATCTGCCCTAAG	60.1	136	90.389	0.932
		S4_1351-r2	CCATTAGGTATAAAAAGGGTTGGTTAAGTTGG	61.3			
SNP_IGA_695463	EPPISF002	S6_2645-f2	CTTGTTCACCCGTCGTGGAGGCT	63.1	68	96.83	0.994
		S6_2645-r2	GCACTTCCCAAGGTGGTCGTTTCC	63.5			
SNP_IGA_786935	UDAp-409A	S7_1954b-f1	CAATCCAAAGCTGCTCACCTCCA	60.1	124	102.906	0.986
		S7_1954b-r1	GACCTGGCTCCTGACGGAGTTG	59.6			
SNP_IGA_112592	BPPCT036B	S1_3674-f2	ACAGAGAGGTTCACATTGGCTTTACAAA	59.5	149	92.303	0.960
		S1_3674-r2	GAAGCTGGGTGATAAGTAATTTTCAATAAACAAGCA	64.6			
rs159239801	M12a	S4_9208b-f1	CTGTCTTGGTATCAATCCACTGTGAGACTT	60.1	150	89.051	0.996
		S4_9208b-r1	AGCCAAGTCCAATTTCGTTTCAACTAATG	61.6			
SNP_IGA_780662	UDAp-460	S7_1667-f1	GGTTTCGGTTTCTTCTTCGTCCA	58.2	97	91.915	0.998
		S7_1667-r1	AACGACAAGTCGCATCAGGATCAG	59.2			
		S1_4475-r1	CCAATCCTGACAACTAGCATTGATTGAC	60.0			
1_40995799	1_40995799	S1_4099-f1	CGAACAATCCAACTGGCAGTGC	59.1	96	102.331	0.999
		S1_4099-r1	AGGAGTCATAAACAATTATTGATCCGTTTG	59.1			
2_16900230	2_16900230	S2_1690-f1	CAAATTACAAACAGCCACCTCATCAGC	60.9	114	92.092	0.991
		S2_1690-r1	GTGACCGTCGGATTCGCCAT	59.0			
4_00772820	4_00772820	S4_0077-f1	CATGGTCGTGTTGTCTCTGCATTG	59.0	93	89.503	0.995
		S4_0077-r1	GAGAAACGGTGTTGACTGAGCAGC	59.0			
4_11060745	4_11060745	S4_1106-f1	CCGATTGGTTGATGCTGTGGATC	60.0	133	108.264	0.976
		S4_1106-r1	GAAGTAAAGGTTATCGAAATGGTTTCTCG	59.0			
4_13747914	4_13747914	S4_1374-f1	ACAAGGCTGGGTTGTAGGCTGC	59.2	131	99.112	0.984
		S4_1374-r1	GCTGGATCAGGAGGCAAAATTAGG	59.1			
SNP_IGA_427604	4_14984691	S4_1498b-f2	AATCTACTGAGATTCTAGTATGAGAGAGGTCTAAGC	58.9	134	99.598	0.982
		S4_1498b-r2	CATTTTCCACCCACCAAACCTTCGAC	64.1			
5_13713689	5_13713689	S5_1371-f2	CACTCTGAATCCTTCTGTTGGGTTGGC	63.7	132	92.307	0.992
		S5_1371-r2	AATATCAGTGCAGCTTTCAGGGACAAGAAG	62.8			
8_11718744	8_11718744	S8_1171-f1	CATGGAGATCAGTAATGAAACATCTCTGC	59.4	96	96.596	0.998
		S8_1171-r1	GCCCACTGACAGCTTCTTCAACC	58.8			

**^1^** Selected marker: the flanking SNP marker representing the original cofactor as the selected SNP marker. ^2^ The original cofactor linked with QTL. ^3^ Ta: annealing temperature. ^4^ Efficiency of PCR and R^2^ were determined by the optimal standard curves constructed using the Ct value and genomic DNA quantity. Specifically, peach leaf genomic DNA was serially diluted with deionized water and used as template for qPCR. PCR efficiency was calculated with StepOnePlus software v. 2.3 with a formula of PCR efficiency (PCR efficiency = 10^−1/^slope ^– 1^). Technical repeats = 2.

**Table A3:** Genotypes of the selected SNP markers linked to minor-effect CR-related QTLs in 27 peach cultivars.

Cultivar	CR (h)	SNP_IGA_297497	rs159238319	SNP_IGA_695463	SNP_IGA_112592	SNP_IGA_419106	rs159239801	2_16900230	4_00772820	4_11060745	5_13713689	8_11718744
**Low-chill cultivars**												
Okinawa	100	C^†^	C	T	A	T^†^	T	T/G	A	G	C^†^	T^†^
Flordared	100	C	C	T	A^†^	T/C	T/G	G^†^	A/G^†^	C/G	C	C^†^
Ruby	100	C	T/C^†^	G	G	C	T/G	T	A/G	C/G	C	C
Xiami	125	C	C	T	A	T/C	T/G^†^	G^†^	A/G	C/G	C^†^	C^†^
Yinggetao	125	C	C	T^†^	G	C	T^†^	T	A	G	C	C^†^
Premier	150	T/C	T/C^†^	T/G	A/G	T	T	T/G	A/G	C/G	C	T/C^†^
Flordabell	150	T	C^†^	T^†^	A/G	C^†^	T/G^†^	T/G^†^	A/G	C/G^†^	C^†^	C
Flordabeauty	150	T/C	C	T/G	A/G^†^	T/C^†^	T/G^†^	T^†^	A/G	C/G	C	T/C^†^
TropicPrince	150	T/C	C	T/G	A/G^†^	T^†^	T^†^	T^†^	A	G^†^	C/G^†^	T
Kuu Taur	150	C	C	T	A	C^†^	T	T^†^	A^†^	G	C	T^†^
Chuenfeng	150	C	T/C^†^	T^†^	A/G	T/C	T/G	G	G^†^	C/G	C	T/C
TropicSweet	175	C	C^†^	T/G^†^	A/G^†^	T/C	T/G	T^†^	A^†^	C/G	C	T/C^†^
SpringHoney	180	C	T/C^†^	T/G	G	T/C	T	T/G	A/G	C/G	C	T/C^†^
Tropicsnow	200	C	C^†^	G	A	T^†^	T/G^†^	T/G	A/G	C/G	C	C
Fushou	N/A^5^	C	C	T	G^†^	T/C^†^	T^†^	T/G	A^†^	C/G	C/G^†^	C^†^
**High-chill cultivars**												
Yamane Hakuto	800	T/C	C	G	A	C	T	T/G	A	C/G^†^	C	T
Shiga Hakuto	800	T	C	G	A^†^	T/C^†^	T^†^	T	A	C/G^†^	C	T
Okubo	850	T^†^	C	T/G	A^†^	C	T	T/G	A	C/G	C	T^†^
Shang Hai Shui Mi	850	T	C	T^†^	A^†^	T	T	T/G	A	C/G^†^	C/G^†^	T^†^
Okitsu	900	T/C	C	T	G^†^	T	T	T/G	A/G	G	C^†^	T
Aki Hakuto	900	T	C	T/G	A	C	T	T/G	A	G	C	T
Hongqingshui	N/A	T	C	T/G	A	C	T^†^	T^†^	A/G^†^	C/G	C/G^†^	T
Nakatsu Hakuto	N/A	T/C	C^†^	G^†^	A	C	T	T/G	A^†^	C/G	C	T^†^
Sunago wase	N/A	T/C	C	T	A	T/C^†^	T	T/G	A	C/G	C^†^	T
Yamato Wase	N/A	T/C^†^	C	G	A	C	T	T/G	A/G^†^	C/G^†^	C	T^†^
Odama Hakuho	N/A	T/C	C	G	A	C	T	T/G	A^†^	C/G^†^	C	T^†^
Tsao Sheng Yu Tao	N/A	C	T/C^†^	T	A	T	T^†^	T/G	G	G	C	T
Putative low chill associated marker		C	--	T	G	T	T/G	--	G	--	--	C
Significance (*X^2^*-test) ^4^		**	ns.	ns.	**	ns.	**	ns.	ns.	ns.	ns.	***
Accuracy (ratio; %)		3/3; 100%	8/9; 88.9%	5/6; 83.3%	9/9; 100%	6/9; 66.7%	10/10; 100%	5/8; 62.5%	9/9; 100%	7/7; 100%	9/9; 100%	15/15; 100%

**^1^** Cultivar name: for cultivars without English names, the names were transliterated based on the pronunciation of their Japanese or Chinese name. ^2^ Putative low chill specific markers: the SNP sequence variant is relatively more frequent in low-chill cultivars than in high-chill cultivars. ^3^ The accuracy of each marker was calculated as follows: the number of variant calls consistent with Sanger sequencing / total sample number sequenced with Sanger sequencing in ratio and percentage. ^4^ Association analysis between genetic markers and peach CR traits using a contingency table chi squared-test (*X^2^*). * *p* < 0.05, ** *p* < 0.01, and *** *p* < 0.001. ns.: not significant. ^5^ N/A: not available. ^†^ The genotyping results validated with the Sanger sequencing method.
